# Identification of RNA reads encoding different channels in isolated rat ventricular myocytes and the effect of cell stretching on *L-type Ca*^*2+*^*current*

**DOI:** 10.1186/s13062-023-00427-0

**Published:** 2023-10-30

**Authors:** Andre G. Kamkin, Olga V. Kamkina, Viktor E. Kazansky, Vadim M. Mitrokhin, Andrey Bilichenko, Elizaveta A. Nasedkina, Stanislav A. Shileiko, Anastasia S. Rodina, Alexandra D. Zolotareva, Valentin I. Zolotarev, Pavel V. Sutyagin, Mitko I. Mladenov

**Affiliations:** 1 Department of Physiology, Pirogov Russian National Research Medical University, Moscow, Russian Federation; 2 Faculty of Natural Sciences and Mathematics, Institute of Biology, “Ss. Cyril and Methodius” University, Skopje, North Macedonia

**Keywords:** Rat ventricular myocytes, Mechanosensitive cation channels, Stretch-activated channels, mRNA, Transcriptomic profile, Ca_V_1.2 channels

## Abstract

**Background:**

The study aimed to identify transcripts of specific ion channels in rat ventricular cardiomyocytes and determine their potential role in the regulation of ionic currents in response to mechanical stimulation. The gene expression levels of various ion channels in freshly isolated rat ventricular cardiomyocytes were investigated using the RNA-seq technique. We also measured changes in current through Ca_V_1.2 channels under cell stretching using the whole-cell patch-clamp method.

**Results:**

Among channels that showed mechanosensitivity, significant amounts of TRPM7, TRPC1, and TRPM4 transcripts were found. We suppose that the recorded *L-type Ca*^*2+*^ current is probably expressed through Ca_V_1.2. Furthermore, stretching cells by 6, 8, and 10 μm, which increases *I*_SAC_ through the TRPM7, TRPC1, and TRPM4 channels, also decreased *I*_Ca,L_ through the Ca_V_1.2 channels in K^+^ _in_/K^+^ _out_, Cs^+^ _in_/K^+^ _out_, K^+^ _in_/Cs^+^ _out_, and Cs^+^ _in_/Cs^+^ _out_ solutions. The application of a nonspecific *I*_SAC_ blocker, Gd^3+^, during cell stretching eliminated *I*_SAC_ through nonselective cation channels and *I*_Ca,L_ through Ca_V_1.2 channels. Since the response to Gd^3+^ was maintained in Cs^+^ _in_/Cs^+^ _out_ solutions, we suggest that voltage-gated Ca_V_1.2 channels in the ventricular myocytes of adult rats also exhibit mechanosensitive properties.

**Conclusions:**

Our findings suggest that TRPM7, TRPC1, and TRPM4 channels represent stretch-activated nonselective cation channels in rat ventricular myocytes. Probably the Ca_V_1.2 channels in these cells exhibit mechanosensitive properties. Our results provide insight into the molecular mechanisms underlying stretch-induced responses in rat ventricular myocytes, which may have implications for understanding cardiac physiology and pathophysiology.

**Supplementary Information:**

The online version contains supplementary material available at 10.1186/s13062-023-00427-0.

## Background

During the last five decades, scientists have collected a lot of evidence that mechanical stress has a significant impact on the electrophysiological properties of cardiomyocytes. This phenomenon, commonly referred to as mechanoelectric feedback, has been extensively studied [1] and is believed to play a crucial role in the pathophysiology of cardiac arrhythmias [2, 3]. In healthy hearts, this feedback mechanism can involve transmembrane cation fluxes through stretch-activated channels (SACs) [4], which can modulate the membrane potential of cardiac myocytes [5–7]. Scientific data have shown that localized stretching of single ventricular or atrial myocytes involves cation flux through cation-nonselective SACs [5], which in turn can affect the membrane potential of these cells. Stretch sensitivity is particularly high in hypertrophied ventricular cardiomyocytes from spontaneously hypertensive rats and in atrial and ventricular cardiomyocytes from patients with end-stage heart failure [8]. In general, the role of cardiomyocyte SACs is not limited to normal heart function but also plays a crucial role in various pathological conditions.

Several studies reported Ca^2+^ as well as Na^+^ and K^+^ permeability in SACs of tissue-cultured embryonic chick cardiac myocytes [9–12] and rat atrial myocytes [10]. Mechanical stretching has been suggested to increase the amplitude of Ca^2+^ transients by activating SACs, which, in turn, increases their Ca^2+^ permeability [12, 13].

Although the L-type Ca^2+^ channel is considered the main pathway of Ca^2+^ entry during an action potential (AP) in cardiomyocytes [14, 15], there are several studies showing that uniaxial stretching does not affect the *L-type Ca*^*2+*^ current (*I*_Ca,L_) in single cardiomyocytes [16–19]. However, in our previous studies, we observed inhibition of *I*_Ca,L_ during uniaxial stretching using a glass stylus [6, 8]. This inhibition was attributed to the intracellular accumulation of Ca^2+^ due to the Ca^2+^ influx through SACs. To date, no studies have been conducted that examined the dependence of the value of *I*_Ca,L_ on the degree of axial cell stretching. Although there may be no significant changes in *I*_Ca,L_ by uniaxial stretch in a voltage clamp experiment, extra Ca^2+^ may still enter the cell through L-type Ca^2+^ channels during prolongation in the depolarization phase of AP due to delayed voltage-dependent inactivation, which partly contributes to the increase in the amplitude of the *Ca*^*2+*^*transients* [6, 20]. To examine the effects of cell stretching on L-type Ca^2+^ channels in rat ventricular myocytes, we investigated the operation of these channels under different degrees of dosed stretching.

In addition, to understand the function of operative SACs and Ca^2+^ channels, RNA reads encoding all existing channels in adult rat ventricular myocytes were examined. The data obtained showed the maximum number of RNA reads for the Ca_V_1.2 channels, which exhibit additional mechanosensitive properties. Among mechanosensitive channels, we identified significant amounts of transcripts for transient receptor potential ion channels, including (melastatin) TRPM7, (canonical) TRPC1, and TRPM4.

## Materials and methods

### Animals

The experiments were conducted in compliance with the Guide for the Care and Use of Laboratory Animals (8th edition, 2011) published by the US National Institutes of Health, and the experimental protocol was approved by the Ethics Committee of the Russian National Research Medical University. Male outbred Wistar rats (n = 34) weighing between 220 and 270 g (10–12 weeks) were housed in a standard T4 cage in a 12:12 h light : dark cycle and given *ad libitum* access to food for four weeks prior to the start of the experiment.

### Solutions

Ca^2+^-free physiological salt solution (Ca^2+^-free PSS) containing (in mM: 118 NaCl, 4 KCl, 1 MgCl_2_, 1.6 NaH_2_PO_4_, 24 NaHCO_3_, 5 sodium pyruvate, 20 taurine, and 10 glucose, adjusted to pH 7.4 with NaOH (bubbled with carbogen 95% O_2_ + 5% CO_2_). Enzyme medium containing: Ca^2+^-free PSS supplemented with 10 µM CaCl_2_, 0.2 mg ml^− 1^ collagenase (Type II, Worthington, 225 U mg^− 1^), and 1 mg ml^− 1^ bovine serum albumin (Sigma). Before the actual experiments, cells were stored for at least 2 h in a modified Kraftbrühe (KB) medium, containing (mM): 50 L-glutamic acid, 30 KCl, 3 MgSO_4_ × 7H_2_O, 20 taurine, 10 glucose, 30 KH_2_PO_4_, 0.5 EGTA, and 20 HEPES, adjusted to pH 7.3 with KOH [6]. The isolated cells were stored in KB medium for up to 8 h. Ventricular cardiomyocytes were perfused with a solution (37 ^o^C) containing (mM): 150 NaCl, 5.4 KCl, 1.8 CaCl_2_, 1.2 MgCl_2_, 20 glucose, and 5 HEPES, at pH 7.4, adjusted with NaOH (K^+^ _out_ solution). Internal pipette solution containing (mM): 140 KCl, 5 Na_2_ATP, 5 MgCl_2_, 0.01 EGTA and 10 Hepes/KOH at pH 7.3 (K^+^ _in_ solution). Later in the text, this configuration is referred to as K^+^ _in_/K^+^ _out_ solutions. In some experiments, we suppressed *inward rectifying K*^*+*^ currents by substituting Cs^+^ for extracellular K^+^ and reduced *outward rectifying K*^+^ currents by replacing K^+^ with Cs^+^ in the intracellular (electrode) solution. Below in the text, this configuration is referred to as Cs^+^ _in_/Cs^+^ _out_ solutions. For this, in a perfusion solution, 5.4 mM KCl was replaced by 5.4 mM CsCl (extracellular Cs^+^_оut_ solution), while in the patch pipette solution, 140 mM KCl was replaced by 140 mM CsCl (intracellular Cs^+^ _in_ solution) [5]. With suppressed *K*^*+*^ currents, the peak *L-type Ca*^*2+*^ current can be approximated by subtracting the late current (*I*_*L*_) from the most negative current surge [21]. A similar substitution was made to separate the effects of non-selective cation currents *I*_ns_ [5, 6].

### Isolated cardiomyocyte preparation

We followed a modified version of the previously described cell isolation procedure [5, 6]. Male Wister rats were anesthetized with an intraperitoneal injection of 80 mg kg^-1^ ketamine and 10 mg kg^-1^ xylazine, with heparin (1000 U kg^-1^) added to the anesthetic solution to prevent blood coagulation in the coronary vessels of the excised heart. The chest was opened, and the heart was rapidly excised and attached to a Langendorff apparatus with a constant flow of 1 ml min^-1^ at 37 °C to flush the coronary vessels in carbogen-bubbled Ca^2+^-free PSS for 5 min. After this initial perfusion, hearts were retrogradely perfused with the same PSS supplemented with Worthington type II collagenase (0.5 mg ml^-1^), 1 mg ml^-1^ bovine serum albumin (Sigma), and 10 µM CaCl_2_ for 18–20 min. The perfusate was continuously bubbled with carbogen (95% O_2_ + 5% CO_2_), and the temperature was equilibrated at 37 °C. The enzymes were then washed out with a modified KB medium [22], and the heart was disconnected from the perfusion system. The ventricles were then cut off, chopped, and gently triturated to release the cells into the KB medium. The resulting cell suspension was filtered and stored in KB medium at 22 °C prior to use.

### mRNA isolation and purification

mRNA was isolated directly from cardiomyocytes using Oligo-dT25 Dyna beads® (Invitrogen). The mRNA-bound beads were washed in a Tris buffer solution containing lithium dodecyl sulfate (LiDS) or without LiDS (Buffer A: 10 mM TrisHCl (pH 7.5); 0.15 M LiCl; 1 mM EDTA; 0.1% LiDS; Buffer B: 10 mM TrisHCl (pH 7.5); 0.15 M LiCl; 1 mM EDTA). The beads were resuspended in RNase-free water (Sigma, Dorset, UK), and the mRNA was reverse transcribed using cloned AMV reverse transcriptase (Invitrogen). The resulting cDNA was stored at -80 °C until necessary.

### RNA sequencing and analysis

The obtained libraries were analyzed for concentration, size distribution, and quality using a dsDNA high sensitivity kit (Invitrogen, Carlsbad, CA, USA) on a Qubit 4 fluorometer (Thermo Fisher Scientific, Inc., Dreieich, Germany) and a high sensitivity D5000 kit (Agilent, Santa Clara, CA, USA) on a 4200 TapeStation. Libraries were normalized according to their molarity, pooled, and then quantified with a library quantification kit for Illumina platforms (Roche, Basel, Switzerland) using a StepOnePlus qPCR machine (Thermo Fisher Scientific, Inc., Dreieich, Germany). The resulting pooled libraries were loaded at 350 pM with 1% PhiX on an S2 FlowCell and sequenced in triplicate using a NovaSeq 6000 next-generation sequencer (Illumina, San Diego, CA, USA) with paired-end reads of 2 × 150 bp.

Raw FASTQ - sequenced reads were first assessed for quality using FastQC v0.11.5 (available online at http://www.bioinformatics.babraham.ac.uk/projects/fastqc/) [23]. The reads were then passed through Trimmomatic v0.36 [24] for quality trimming and adapter sequence removal. The surviving trimmed read pairs were then processed with Fastp [25] to remove poly-G tails and Novaseq/Nextseq-specific artifacts. Following the quality trimming, the reads were assessed again using FastQC. Post - QC and QT, the reads were aligned to the human reference genome GRCh38.p4 using HISAT2 [26] with default parameters, and the resulting SAM alignments were then converted to BAM format and coordinate sorted using SAM Tools v1.3.1 [27]. Finally, the sorted alignment files were analyzed with HTSeq-count v0.6.1p1 [28] using the options (-s no -t exon -I gene_id) for raw count generation.

Unless otherwise stated, all experiments were performed with at least three replicates, and the data are represented as the mean ± standard error (SE).

### Compounds

In certain experiments, nifedipine (Sigma-Aldrich), a specific blocker of the L-type Ca^2+^ channel [29], was added to the bath solution at a concentration of 5–10 µM to assess the pharmacological specificity of the observed Ca^2+^ currents [30, 31]. At these concentrations, nifedipine selectively blocks *I*_Ca,L_ without affecting the *T-type Ca*^*2+*^ current (*I*_Ca,T_), the *fast Na*^*+*^ current, the *delayed rectifier* current (*I*_*K*_), or the hyperpolarization-activated *inward* current [29]. Isoproterenol, a ß-adrenergic stimulator, dose-dependently stimulated *I*_Ca,L_ in isolated cardiac myocytes, reaching a plateau at 100 nM. The effect of isoproterenol (Sigma-Aldrich) (20 nM) on the peak current amplitudes of *I*_Ca,L_ at 0 mV was considered a test of the quality of isolated cells. Gadolinium (Gd^3+^) (Sigma-Aldrich), known as a non-specific blocker of mechanically gated channels in the *I*_SAC_ (current through stretch activated channels) [5, 6], was added to the PSS (5 µM GdCl_3_), BAPTA (Tocris), and 1,2-bis(2-aminophenoxy)ethane-N,N,N’,N’-tetraacetic acid.

### Mechanical stretch of the ventricular myocytes

The mechanical stimulation method used in this study has previously been described in detail. Here, we only report the peculiarities relevant to this study. After whole-cell access with the patch pipette (P), a fire-polished glass stylus (S) was attached to the membrane [5, 6, 22, 32]. When the stylus was newly polished and the surface membrane was clean, the attachment was successful in approximately 70% of the attempts. The stylus was then lifted 2 μm to prevent ‘scratching’ of the lower cell surface on the coverslip during the stretch. A motorized micromanipulator (MP 285, Sutter, Novato, Calif., USA, accuracy 0.2 μm) increased the S-P distance stepwise by up to 12 μm, with P being the fixed point [5, 22]. Stretch and release of stretch could be repeated 3–4 times with the same cell, on average. Our method was shown to stretch the cell surface locally, while the membrane in the line between P and S was stretched as expected (approximately 80% of the entire membrane surface remains unaffected) [5, 22]. The effect of mechanical stretching on the sarcomere pattern was imaged by a slow-scan CCD camera (Princeton Instruments, Trenton, N.J., USA) and evaluated by Meta Morph software (Universal Imaging, West Chester, PA, USA). S and P were positioned 40 μm apart before attaching them to the cell. Cell stretching by 4 μm (increasing the S - P distance) increased local stretching by approximately 6%, by 6 μm about 10%, by 8 μm about 14%, and by 10 μm about 18%. These values were less than expected but close to those previously obtained in isolated mouse cardiomyocytes. Presumably, the extent of local stretch decays from the cell surface to the interior of the cell, where the optical focus was set [6, 22].

To investigate the effect of cell stretch at 4, 6, 8, and 10 μm on *I*_Ca,L_ values, measurements were taken. A standard elongation of 6 μm was used to study *I*_Ca,L_ on the background of cell elongation under the action of various compounds.

### Whole-cell patch-clamp

A total of 113 cells (n = 113) were used in the experiments. Whole-cell patch-clamp recordings of *K*^*+*^, *Ca*^*2+*^, and non-selective (*I*_ns_) currents were obtained using an Axopatch 200B amplifier and pClamp 10 software (Molecular Devices, San Jose, CA, USA). The data were filtered at 2 kHz, sampled at 5 kHz, and analyzed using the same software. Myocytes were superfused in a small recording chamber (RC-26; Warner Instrument Corp., Brunswick, CT, USA) with a volume of 150 µl, which was mounted on an inverted microscope.

The borosilicate glass patch-clamp electrodes had tip resistances ranging from 1.5 to 2.5 MΩ when filled. Cell access was obtained by rupturing the patch after seal formation. Pulses of 140 ms and 350 ms were applied at 1 Hz, starting from a holding potential (*V*_hp_) of − 45 mV, which caused the inactivation of tetrodotoxin (TTX)-sensitive *Na*^*+*^ currents. To evaluate membrane capacitance and access resistance, currents in response to trains of short (5 mV) pulses applied at − 45 mV were taken without compensation for capacitive and leak currents. Cells with similar geometry were selected based on their length and diameter (control rat ventricular cardiomyocytes had an average diameter of 25 ± 6 μm), and, on average, had a membrane capacitance of 150 ± 16 pF (n = 16) and an input resistance of 58 ± 5 MΩ (in 16 representative cells). Glass tools were adjusted to the same 40 μm S -P distance before the application of stretch to minimize the effect of differences in the size of the stretched membrane. Since the area of mechanical stretching was small and unknown, we did not divide the stretch-induced currents by the entire membrane capacitance.

Currents through L-type Ca^2+^ channels, membrane currents at the end of the pulse (late current: *I*_L_), and other currents were plotted as functions of their respective clamp step potentials. The seal resistance remained constant, i.e. it was 1.5 ± 0.3 GΩ before and 1.4 ± 0.4 GΩ during the stretch. Similarly, access resistance and membrane capacitance remained unaffected, indicating that the stretch-induced inward current was due to the activation of an ionic current rather than leakage around the seal. The zero current potential (*V*_0_) for *I*_L_ was determined by the intercept of the resulting *I/V* curve with the voltage axis and corresponded to the resting membrane potential of a non-clamped cell (between − 70 and − 80 mV). Online records of net membrane current were carried out at *V*_hp_ = − 45 mV (time course) [6, 22, 33].

Typically, the measurements lasted approximately 30 min, during which time the access resistance and capacitive current remained stable. To obtain the current-voltage relations (*I/V* curves), a series of 20 pulses of 140 ms (or 350 ms) duration were applied, starting from *V*_hp_ = − 45 mV.

Freshly isolated brick-like cardiomyocytes can attach to the glass bottom in two different positions: edgewise, staying on the narrow side, and broadwise, staying on the broad side [34]. However, the response to stretching is identical in cardiomyocytes occupying both positions (edgewise and broadwise). On the other hand, the response to compression differs depending on the cell’s position [34, 35]. For our experiments, we selected cells that stayed on the narrow side (edgewise) and had similar sizes.

In this study, *I*_Ca,L_ recordings are presented in both absolute values (e.g., pA) and normalized form (e.g., pA/pF). However, changes in *I*_Ca,L_ in response to cell stretching or treatment with compounds are presented as the difference between *I*_Ca,L_ under control conditions and that under stretching or treatment conditions.

### Statistics

Values are presented as means ± SD. To determine significant differences, we used Analysis of Variance (ANOVA) with the Bonferroni test as a post-hoc analysis. For cases where multiple factors were evaluated, a two-way ANOVA was performed. Statistical significance was set at p < 0.05.

## Results

### The levels of the channel’s gene expression

**Fig. 1:**
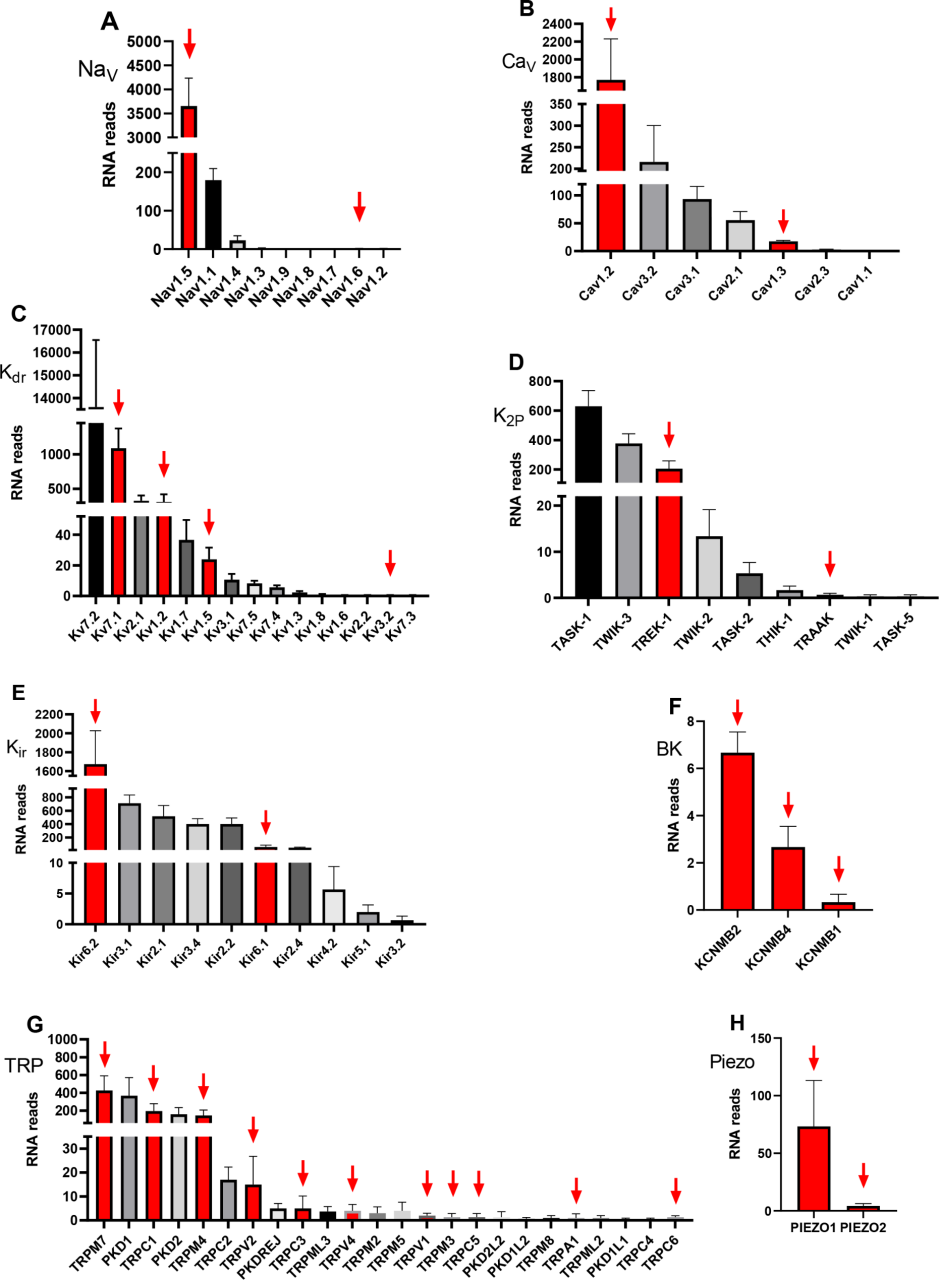
Relative abundance of ion channels encoding different channel genes in adult rat ventricular cardiomyocytes Bars represent the mean ± SEM (n = 10). Red columns and red arrows indicate mechanosensitive channels, while black and gray columns indicate all the rest

In this study, we used RNA-seq to measure the expression levels of various genes in freshly isolated rat ventricular cardiomyocytes. Our analysis revealed the presence of RNA reads encoding different ion channels, as summarized in Table 1 [36–92], and illustrated in Fig. 1. Specifically, we found that Na_V_ channel transcripts were expressed in these cells, with Na_V_1.5 showing the highest number of RNA reads (3654.4 ± 581.2), followed by Na_V_1.1 (only 179 ± 30), [81, 82]. The expression of other Na_V_ channels was minimal. Importantly, previous research has shown that Na_V_1.5 exhibits mechanosensitivity [81, 82], which may have physiological implications for the function of rat ventricular cardiomyocytes.

**Table 1:** Identified mechanically gated channels and channels with mechanosensitivity in our experiments and in the literature

Ion channels	Species	Material for analysis	Number of reads mapped	Number of tests (n)	Known data		
					Species	Location	Mechanosensitivity
**Mechanically gated channels**: *Non-selective cation channels*							
TRPM7	Rat	Isolated ventricular myocytes	333.5 ± 31.5	3	Pig and Human	Atrial tissues [36]	The authors did not investigate
					Human TRPM7	TRPM7 channel expressed in HeLa cells	TRPM7 is a stretch-activated channel [37].
					Human TRPM7	TRPM7 channel expressed in HEK293T cells	TRPM7 single channels are directly activated by stretch [38].
					Human TRPM7	TRPM7 channel expressed in vascular smooth muscle A7R5 cells	TRPM7 is directly activated by shear stress [39]
TRPC1	Rat	Isolated ventricular myocytes	146.66 ± 6.96	3	Human	Heart [40]	The authors did not investigate
					Rat	Atrial myocyte, ventricular myocyte, Purkinje cells	TRPC1 is a stretch-activated channel [40, 41]
					Human TRPC1	Human TRPC1 transfected into CHO-K1 cells	TRPC1 mediate stretch-activated currents [42]
					Human TRPC1	TRPC1 channel expressed in oocytes *Xenopus laevis*	TRPC1 is a stretch-activated channel [43]
					Human TRPC1	Human TRPC1 transfected into CHO-K1 cells	TRPC1 is a mechanosensitive channel [43]
TRPM4	Rat	Isolated ventricular myocytes	179.66 ± 5.93	3	Human	Purkinje fibers, atrial cardiomyocytes, ventricular cells [44]	The authors did not investigate
					Rat	Freshly isolated cardiomyocytes from the left ventricles [45]	The authors did not investigate
					Human	Human TRPM4 transfected into HEK293 cells [46]	TRPM4 is a mechanosensitive channel [46, 47].
					Rat	Cerebral artery myocytes [47]	
TRPV2	Rat	Isolated ventricular myocytes	21.66 ± 2.03	3	Mice	Isolated cardiomyocytes [48]	The authors did not investigate
					Mice	Freshly dispersed vascular smooth muscle aortic myocytes	TRPV2 is a mechanosensitive channel [49].
TRPC3	Rat	Isolated ventricular myocytes	5.00 ± 3.00	3	Human	Heart [40]	The authors did not investigate
					Mice	Ventricular myocytes [50]	
					Rat	Ventricular myocytes [51]	
					Mice	Ventricular myocytes [52]	
					Mice	Ventricular myocytes	TRPC3 is a stretch-activated channel [53]
					Canis	Cardiac atrial fibroblasts	TRPC3 is a stretch-activated channel [54]
						TRPC3 expressed in NDC cells or CHO	TRPC3 is a stretch-activated channel [55]
TRPV4	Rat	Isolated ventricular myocytes	5.5 ± 0.50	3	Neonatal rat	Cultured ventricular myocytes	TRPV4 is a stretch-activated channel [56]
					Rat TRPV4	TRPV4 channel expressed in oocytes *Xenopus laevis*	TRPV4 is a stretch-activated channel [57].
					Human	Heart [40]	The authors did not investigate
					Murine TRPV4	The TRPV4 channel expressed in vitro in Chinese hamster ovary cells	TRPV4 is a mechanosensitive channels [58].
TRPV1	Rat	Isolated ventricular myocytes	2.00 ± 0.58	3	Rat	Kidney. Cloned from the rat kidney	TRPV1 is a mechanosensitive channel (initially named “stretch-inactivated channel”), [59].
					Mice	Bladder urothelium	TRPV1A is a mechanosensitive channel [60].
TRPM3	Rat	Isolated ventricular myocytes	1.33 ± 0.88	3	Human, bovine, mice	TRPM3-expressing HEK-293 cells (Expression in bovine and human kidney; in mouse and human brain).	TRPM3 is a mechanosensitive channel [61]
TRPC5	Rat	Isolated ventricular myocytes	1.33 ± 0.88	3	Human	Heart [40]	The authors did not investigate
					Murine TRPC5	TRPC5 channel expressed in HEK-293 cells	TRPC5 is a stretch-activated channel [62]
TRPA1	Rat	Isolated ventricular myocytes	1.0 ± 0.9	3	Droso-phila	Heart [63]	TRPA1 is a mechanosensitive channel [63]
					Mice	Inner ear hair cells	TRPA1 is a mechanosensitive channel [64]
						*Caenorhabditis elegans*	TRPA1 is a mechanosensitive channel [65]
TRPC6	Rat	Isolated ventricular myocytes	1.0 ± 1.0	3	Human	Heart [40]	The authors did not investigate
					Mice	Isolated ventricular myocytes	TRPC6 is a stretch-activated channel [22]
					TRPC6	HEK-293 cells were transfected with the TRPC6 vector	TRPC6 is a mechanosensitive channel [66]
					Cerebral artery	Myocytes	TRPC6 is a mechanosensitive channel [47]
					Heart	Ventricular myocyte	TRPC6 is a mechanosensitive channel [67]
PKD1 (Polycys-tin-1, TRPP1)	Rat	Isolated ventricular myocytes	267.33 ± 17.9	3	Mice	Vascular smooth muscle cells	TRPP1 (PKD1) is a mechanosensitive ion channel [68]
PKD2 (Polycys-tin2, TRPP2)	Rat	Isolated ventricular myocytes	191.66 ± 35.09	3	Heart	Ventricular myocyte	TRPP2 (PKD2) is a mechanosensitive ion channel [69, 70]
Piezo 1	Rat	Isolated ventricular myocytes	145.33 ± 8.01	3	Human Piezo1	Piezo1 transfected into HEK-293 cells	Piezo1 is a mechanosensitive channel [71]
					Adult human	Adult human ventricular cardiomyocyte AC16	Piezo1 is a stretch-activated channel [72]
					Rat	Piezo1 channels were natively expressed in dorsal root ganglia tissue.	Piezo1 is a mechanosensitive channel [73]
Piezo 2	Rat	Isolated ventricular myocytes	6.0 ± 2.0	3	Rat	Piezo2 channels were natively expressed in rat dorsal root ganglia tissue.	Piezo2 is a mechanosensitive channel [73]
					Mice	Vagal sensory nerves	Piezo2 is a mechanosensitive channel [74]
**Mechanically gated channels**: *MGCh*_*K*_							
TREK-1/K_2P_2.1	Rat	Isolated ventricular myocytes	153,00 ± 11.93	3	Rat	Isolated ventricular myocyte	TREK-1 is a stretch-activated channel [75]
					Rat	Ventricular myocyte	TREK-1 is a mechanosensitive channel [75]
					Human	Atrial and ventricular tissue.	TREK-1 (K_2P_2.1) is a mechanosensitive channel [76]
					Murine TREK-1	TREK-1 transfected into COS cells	TREK-1 is a mechano-gated K^+^ channel [77]
TRAAK/K_2P_4.1	Rat	Isolated ventricular myocytes	0.66 ± 0.33	3	Human and murine heart	Atrial and ventricular tissue	TRAAK (K_2P_4.1) is a stretch-activated channel [76]
					TRAAK/ K_2P_4.1	TRAAK transfected into COS cells	TRAAK (K_2P_4.1) is a stretch-sensitive K_2_P channel [76]
TREK-2/ K_2P_10.1	Rat	Isolated ventricular myocytes	0	3	Human, Mice	Heart: atrial and ventricle	Stretch-activated cardiac K_2P_ channels [76]
SAKCA or BK_Ca_. Different Types: KCNMB2 KCNMB4 KCNMB1					Chick	Ventricular myocyte	SAKCA is a mechanosensitive channel [78]
					SAKCA	SAKCA cloned from the heart	SAKCA is a mechanosensitive channel [79]
KCNMB2 (BK_Ca_)	Rat	Isolated ventricular myocytes	6.66 ± 0.88	3		The authors did not investigate	The authors did not investigate
KCNMB4 (BK_Ca_)	Rat	Isolated ventricular myocytes	2.66 ± 0.88	3			
KCNMB1 (BK_Ca_)	Rat	Isolated ventricular myocytes	0.33 ± 0.33	3			
K_ATP_					Neonatal and adult rat	Atrial myocyte	K_ATP_ is a mechanosensitive channels [80]
					Rat	Ventricular myocytes	K_ATP_ are mechanosensitive channels [41]
**Voltage-gated channels** with mechano-sensitivity *VGCh*_*MS*_							
Na_V_1.5	Rat	Isolated ventricular myocytes	3158.0 ± 405.7	3		Na_V_1.5 channel expressed in HEK 293 cells	Na_V_1.5 is a mechanosensitive channel [81]
					Human heart Na_V_1.5	Na_V_1.5 channel expressed in *Xenopus laevis* oocytes	Na_V_1.5 is mechanosensitive channel [82]
Na_V_1.6	Rat	Isolated ventricular myocytes	1.0 ± 1.0	3	Mouse Na_V_1.6	Na_V_1.6 channel expressed in *Xenopus laevis* oocytes	Na_V_1.6 is mechanosensitive channel [83]
Ca_V_1.2 L-type	Rat	Isolated ventricular myocytes	1336,66 ± 71.8	3	Ca_V_1.2 subunits	Ca_V_1.2 subunits transfected into HEK-293	Cloned Ca_V_1.2 channel exhibits mechanosensitive behavior similar to the native channel [84]
Ca_V_1.3 L-type	Rat	Isolated ventricular myocytes	17.33 ± 2.03	3	Mice	Inner hair cells (IHCs)	Ca_V_1.3 is mechanosensitive channel [85]
Ca_V_1.1 L-type	Rat	Isolated ventricular myocytes	0.33 ± 0.33	3		The authors did not investigate	The authors did not investigate
Ca_V_2.2 N-type	Rat	Isolated ventricular myocytes	0	3	Human	Human embryonic kidney T-antigen-transformed (HEK-tsA201) cells	Patch-stretch whole cell (1) and whole cell inflation (2) [86]
K_V_1.2	Rat	Isolated ventricular myocytes	212.00 ± 10.02	3	K_V_1.2 channels		K_V_1.2 is a mechanosensitive channel [67]
K_V_1.5	Rat	Isolated ventricular myocytes	30.66 ± 1.67	3	K_V_1.5 channel	K_V_1.5 channel expressed in *Xenopus* oocytes	Stretch by suction (negative pressure) via patch pipette [87]
K_V_3.2	Rat	Isolated ventricular myocytes	0.33 ± 0.33	3	K_V_3.2 channels	K_V_3.2 channel expressed in *Xenopus* oocytes	Stretch by suction (negative pressure) via patch pipette [87]
KCNQ					Chick	Ventricular myocytes	KCNQ channel responds to membrane stretch [67]
K_V_7.1 /KCNQ1	Rat	Isolated ventricular myocytes	821.00 ± 77.20	3	KCNQ1/ K_V_7.1 channel	K_V_7.1 channel expressed in *Xenopus* oocytes	KCNQ1 channel responds to membrane stretch [88]
					KCNQ1/ K_V_7.1 channel	K_V_7.1 channel expressed in *Xenopus laevis* oocytes	KCNQ1 channel responds to hypo-osmotic swelling. Voltage clamp (two-electrode) [89]
					KCNQ1/ K_V_7.1 channel	K_V_7.1 channel in CHO cells	Hypotonic swelling. Patch-clamp studies [90]
**Inward rectifier K**^**+**^**-channels** (K_ir_) with mechano-sensitivity							
K_ir_6.2	Rat	Isolated ventricular myocytes	1240.66 ± 57.1	3	Human Kir6.2	Human Kir6.2 transfected into HEK293T	Kir6.2 is a mechanosensitive only with SUR2A subunit [91]
K_ir_6.1	Rat	Isolated ventricular myocytes	82.66 ± 6.49	3	K_ir_6.1	Expression of Kir6.1 was analyzed in human valve interstitial cells (VIC).	Kir6.1 is a mechanosensitive channel [92]
Kir2.3	Rat	Isolated ventricular myocytes	0	3	Mice	Ventricular myocytes	Kir2.3 is a mechanosensitive channel [22]

Our analysis revealed the presence of three Ca_V_ subfamilies: Ca_V_1.2, Ca_V_1.3, and Ca_V_1.1, as shown in Fig. 1. Of these, the Ca_V_1.2 channels had the highest number of RNA reads (1336.66 ± 71.8), with the transcripts of the other Ca_V_ channels being an order of magnitude smaller. In particular, previous studies have shown that Ca_V_1.2 and Ca_V_1.3 both exhibit mechanosensitivity [84] (Table 1; Fig. 1). When considering the L-type Ca_V_ channels, which are responsible for the *L-type Ca*^*2+*^ current, we found that the Ca_V_1.2 transcripts were significantly more abundant than the Ca_V_1.3 transcripts (17.33 ± 2.03), while the number of Ca_V_1.1 transcripts was very low (Table 1). Based on these results, we conclude that Ca_V_1.2 is likely the primary channel that contributes to the *L-type Ca*^*2+*^ current in rat ventricular cardiomyocytes and that Ca_V_1.3 and Ca_V_1.1 can be neglected. This finding is consistent with previous research indicating that Ca_V_1.2 channels are the main contributors to the *L-type Ca*^*2+*^ current [84].

Among the multitude of K_V_ channel transcripts (Fig. 1), the K_V_7.1 or KCNQ1 channel had the highest number of RNA reads (821.00 ± 77.20), consistent with previous studies showing its response to membrane stretch [88] and hypo-osmotic swelling [88, 89] (Table 1). The K_V_1.2 channel, which has also been shown to exhibit mechanosensitivity [67], was present in smaller quantities with 212.00 ± 10.02 RNA reads (Table 1; Fig. 1). In particular, the number of K_V_1.2 transcripts was 3.5 times less than that of the predominant K_V_7.1 (Fig. 1).

Among K_2_P channels, TREK-1/K_2_P2.1 had the highest number of RNA reads (153.00 ± 11.93) compared to others, consistent with its established mechanosensitivity [75–77]. TRAAK/K2P4.1 channels also exhibit mechanosensitivity [76, 93], although the number of detected RNA reads was very small (0.66 ± 0.33) (Table 1; Fig. 1). Interestingly, transcripts of the mechanosensitive channels TREK-2/K_2_P10.1, which are expressed in the atrial and ventricular cells of human and murine hearts [76], were not detected in our isolated rat ventricular myocytes.

The RNA-seq technique revealed the presence of several Kir channel transcripts (Fig. 1) in freshly isolated rat ventricular cardiomyocytes. Among them, Kir6.2 (1240.66 ± 57.1) was detected in the highest abundance and demonstrated mechanosensitivity only when associated with the sulfonylurea receptor 2 A (SUR2A) subunit [91] (Table 1; Fig. 1). Additionally, RNA reads were found for Kir6.1 channels (82.66 ± 6.49), which are also mechanosensitive [92]. However, unlike mouse ventricular cardiomyocytes, where Kir2.3 channels have been shown to be mechanosensitive [22], we did not detect any RNA reads for Kir2.3 channels in rat ventricular cardiomyocytes.

We identified three types of stretch-activated K_Ca_ (SAKCA), or BK_Ca_ channels, that exhibit mechanosensitivity [78]: calcium-activated potassium channel subunit beta-2 (KCNMB2) (6.6 ± 0.9), KCNMB4 (2.7 ± 0.9), and KCNMB1 (0.3 ± 0.3) (Table 1; Fig. 1). Although ATP-sensitive potassium channels (K_ATP_) also exhibit mechanosensitivity in rats [41, 80], we did not investigate their presence in our study. Likewise, we did not analyze the expression of Kv7 (KCNQ) channels [88–90].

We observed several transcripts for nonselective cation TRP channels, many of which are known to be mechanically gated (listed in descending order of RNA reads). In particular, mechanosensitive TRPM7 (333.5 ± 31.5) [36–39, 94], TRPC1 (146.66 ± 6.96) [40, 42, 43], TRPM4 (179.66 ± 5.93) [44–47, 95], TRPV2 (21.66 ± 2.03) [48, 49], TRPC3 (5.0 ± 3.0) [50–55, 96], TRPV4 (5.5 ± 0.5) [56–58], TRPV1 (2.00 ± 0.58) [59, 60], TRPM3 (1.3 ± 0.9) [61], TRPC5 (1.3 ± 0.9) [62, 97], TRPA1 (1.0 ± 0.9) [63–65], and TRPC6 (1.0 ± 1.0) [22, 47, 66, 67, 97] were among the most abundant.

We also identified two additional stretch-activated channels, Piezo 1 (145.33 ± 8.01) [71–73, 98, 99], and Piezo 2 (6.0 ± 2.0) [73, 74], that were present in the ventricular cardiomyocytes of rats.

The data presented here and below reveal an intriguing observation: While many ion channels have been studied in terms of their response to membrane stretch, only a few have been thoroughly investigated. However, among those that have been studied, all demonstrate a certain degree of mechanosensitivity. On the basis of this, it is plausible to speculate that mechanosensitivity is a ubiquitous characteristic of ion channels and that all channels, irrespective of their gating mechanism, should respond to membrane stretch and tension.

### **Definition and analysis of *L-type Ca***^***2*+**^**current**

**Fig. 2:**
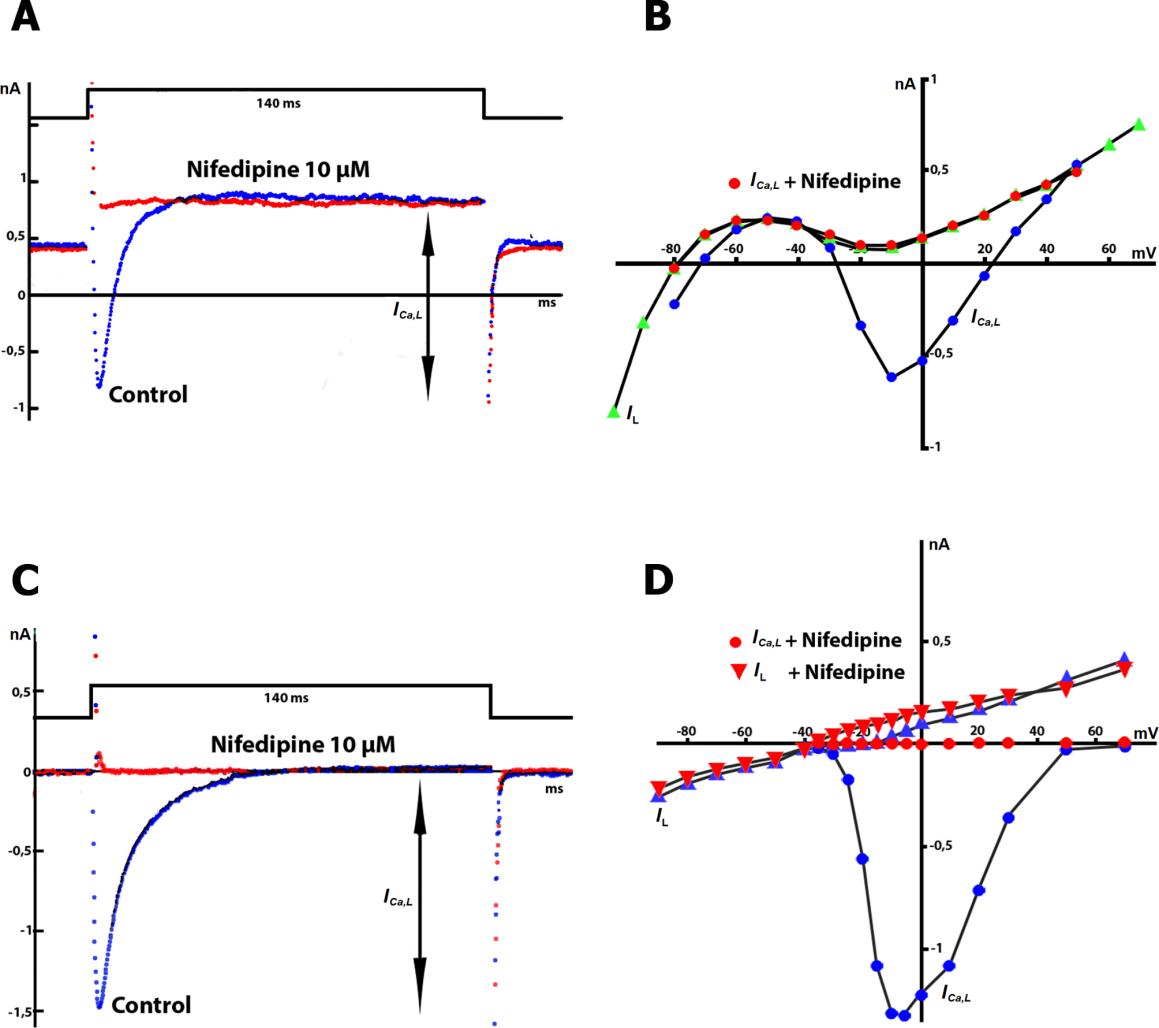
Evaluation of *I*_*Ca,L*_ and *I*_*ns*_ in K^+^ _in_/K^+^ _out_ and Cs^+^ _in_/Cs^+^ _out_ solutions. The membrane potential (*V*_*m*_) was maintained at *V*_hp_ = − 45 mV. **A**: Raw recordings of *I*_L_ and *I*_Ca,L_ (blue trace) and recordings in the presence of nifedipine (red trace) in K^+^ _in_/K^+^ _out_ solutions. **B**: Current-voltage (*I/V*) relationships for *I*_L_ (green triangles) and peak *I*_Ca,L_ (blue circles) under control conditions and in the presence of nifedipine (red circles). **C**: Raw recordings of *I*_L_ and *I*_Ca,L_ (blue trace) and recordings in the presence of nifedipine (red trace) in Cs^+^ _in_/Cs^+^ _out_ solutions, where *K*^*+*^ currents are suppressed. **D**: *I/V* curves for *I*_L_ (blue triangles) and *I*_Ca,L_ (blue circles) under control conditions and in the presence of nifedipine (red circles)

In this study, two methods were used to estimate the current through L-type Ca^2+^ channels (*I*_Ca,L_) in K^+^ _in_/K^+^ _out_ solutions. The first method involved evaluating the time course of *I*_Ca,L_ by subtracting the late current at the end of the 140 ms pulse from the negative peak current (Fig. 2A). The second method involved determining the current-voltage (*I/V*) relationship for *I*_Ca,L_ by subtracting the current at the same potential in the presence of 5 or 10 µM nifedipine from the maximum point of the negative peak current (Fig. 2B). The value of *I*_L_ at 0 mV was almost equal to the current in the presence of nifedipine (Fig. 2A). The negative peak current at 0 mV in control conditions (blue track) and in the presence of 10 µM nifedipine (red track), which selectively blocks L-type Ca^2+^ channels, was used to estimate *I*_Ca,L_. *I*_Ca,L_ was calculated by taking the difference between the negative peak current and the current in the presence of nifedipine (or by subtracting the late current at the end of the 140 ms or 350 ms pulse at a frequency of 1 Hz). Since the *I/V* relation of *I*_L_ in control (green triangles) completely coincides with the *I/V* relation for *I*_Ca,L_ in the presence of nifedipine (red circles) in the range from − 80 mV to + 50 mV (Fig. 2B), the calculation method presented in previous study for K^+^ _in_/K^+^ _out_ solutions was applied [21], whereas *I*_Ca,L_ was estimated as the difference between the negative current at − 10 mV (blue circle) and either the current in the presence of nifedipine at − 10 mV (red circle) or the late current at − 10 mV in control (green triangles) (Fig. 2B).

It should be noted that in K^+^ _in_/K^+^ _out_ solutions, the positive peak on the *I/V* relation for *I*_Ca,L_ between − 30 and − 70 mV (Fig. 2B: blue circles) is associated with the positive currents in response to voltage steps in this potential range and was first demonstrated by Josephson and Sperelakis (1982), [100]. However, not all subsequent studies on *I*_Ca,L_ took this positive peak into account.

Figure 2 C shows that in Cs^+^ _in_/Cs^+^ _out_ solutions, the negative peak current at 0 mV in control conditions (blue track) and in the presence of 10 µM nifedipine (red track) were used to estimate *I*_Ca,L_ by taking the difference between the peak current and the current in the presence of nifedipine or the late current at the end of the pulse. Figure 2D shows the *I/V* relations for *I*_Ca,L_ in control conditions (blue circles) and in the presence of nifedipine (red circles). In this case, *I*_Ca,L_ was calculated as the difference between the negative current (blue circles) and the current in the presence of nifedipine (red circles) or when the current equals 0 nA. The *I/V* relations for *I*_L_ measured at the end of the pulse are shown under control conditions (blue triangles) and in the presence of nifedipine (red triangles). Our experiments used an internal pipette solution containing 0.01 mM EGTA, and under these conditions, we measured the *I*_Ca,L_ value when cells were stretched by 4, 6, 8, and 10 μm. Previous studies have shown that the reduction of *I*_Ca,L_, even by a stretch of 10 μm, was no longer observed when the cells were pre-dialyzed with 5 mM BAPTA, i.e., control *I*_Ca,L_ and *I*_Ca,L_ during stretch superimposed [6]. To study *I*_Ca,L_ in the context of cell elongation under the action of various compounds, a standard elongation of 6 μm was used in this study.

As demonstrated earlier in K^+^ _in_/K^+^ _out_ solutions, the late current (*I*_L_) at the end of the pulse reflects the cell’s response to stretching. Stretch-induced changes in *I*_L_ exhibit an outward rectifying voltage dependence with a reversal potential (*E*_rev_) of − 16 mV [5, 6]. To calculate stretch-induced current (*Δ*^*S*^*I*_L_), we take the difference between the control current values (^*C*^*I*_L_) and the current values in the presence of cell stretching (^*S*^*I*_L_) at − 45 or − 80 mV (*Δ*^*S*^*I*_L(−45)_ and *Δ*^*S*^*I*_L(−80)_) [5].

In our earlier work conducted on isolated mouse ventricular cardiomyocytes [5, 6], as well as in studies by our colleagues on the same cells [22], it was found that in K^+^ _in_/K^+^ _out_ solutions, the net current was composed of *I*_ns_ (current through stretch-activated non-selective cation channels), *I*_*K1*_ (inwardly rectifying potassium current), and *I*_o_ (presumably the sum of several outwardly rectifying currents, such as *K*^+^ currents through TREK channels [76, 77] or the outwardly rectifying canonical transient receptor potential-6 (TRPC6) channels [66]. Cell stretch can modify net membrane currents (*Δ*^*S*^*I*_L_) by modulating both *K*^+^ and *I*_ns_ currents [5]. However, our present studies investigating the RNA transcripts of ion channels and their mechanosensitivity reveal significant differences in the channel spectrum of ventricular cardiomyocytes in adult rats (see below).

To distinguish the effects on *I*_*ns*_, we used two methods: first, we eliminated inward rectifying *K*^*+*^ currents by substituting extracellular K^+^ with Cs^+^ and second, we decreased outward rectifying *K*^*+*^ currents by replacing intracellular K^+^ with Cs^+^. In this case, the current through stretch-activated channels (*I*_SAC_) was calculated as the difference between the control current values (^*C*^*I*_ns_) and the current values on the background of cell stretching (^*S*^*I*_ns_) at − 45 or − 80 mV (*I*_SAC(−45)_ and *I*_SAC(−80)_). We used the same approach to measure the differential current in response to specific compounds (*ΔI*_X_) (where “X” represents the compound) at − 45 or − 80 mV [5, 6].

Under our experimental conditions, isolated cardiomyocytes maintained their fundamental properties, as demonstrated by the significant increase in *I*_Ca,L_ peak current amplitudes at 0 mV in response to 20 nM isoproterenol in K^+^ _in_/K^+^ _out_ and Cs^+^ _in_/Cs^+^ _out_ solutions (not shown), which is consistent with previous literature reports for this compound [101].

### **Local stretch increased net currents*I***_L_**and decreased*I***_**Ca,L**_**in K**^**+**^_**in**_**/K**^**+**^_**out**_**solutions (time-course and voltage-dependence)**

**Fig. 3:**
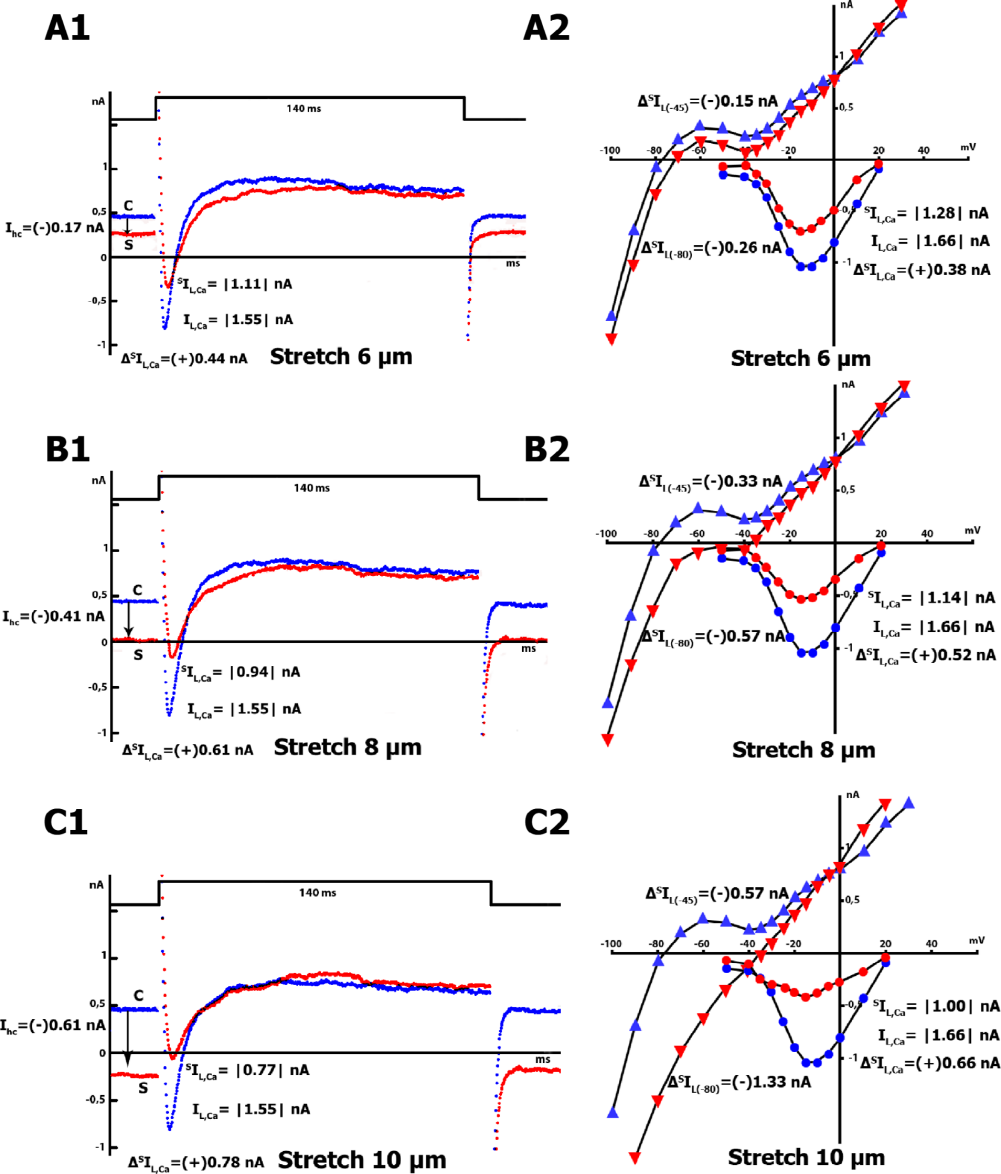
Reduction of *I*_Ca,L_ in K^+^ _in_/K^+^ _out_ solutions during local stretching of cardiomyocytes by 6, 8, and 10 μm. *V*_hp_ = − 45 mV. **A**: (6 μm stretch). **A.1** - The time course of the membrane current. The holding current at *V*_hp_ in control (beginning of the blue traces - label C) and during stretching (beginning of the red traces - label S). A pulse from − 45 to 0 mV induces *I*_Ca,L_, which decreases during stretching (indicated by a negative blue current wave compared to a negative red wave). **A.2** – *I/V* curve of *I*_L_ before (blue triangles) and during (red triangles) stretching, as well as *I*_Ca,L_ before (blue circles) and during (red circles) stretching. **B**: (8 μm stretch). **B.1** - The time course of the membrane current before and during stretching, which results in a greater reduction of *I*_Ca,L_ compared to A.1. Notations as in A.1. **B.2** – *I/V* curves of *I*_L_ and *I*_Ca,L_ before and during stretching. Notations as in A.2. **C**: (10 μm stretch). **C.1** – The time course of the membrane current in control and during stretching. *I*_Ca,L_ decreases with increasing stretching. Notations as in A.1. **C.2** – *I/V* curves of *I*_L_ and *I*_Ca,L_ before and during stretching. Notations as in A.2

Figure 3A.1 illustrates the effect of a 6 μm cell stretch on time-dependent net membrane currents in K^+^ _in_/K^+^ _out_ solutions. At control, the registered holding current (the current at *V*_hp_ = − 45 mV (*I*_hc_)) was + 0.44 nA (label C, Fig. 3A.1), and stretch changed this current (^*S*^*I*_hc_) to + 0.27 nA (label S, Fig. 3A1). The stretch-induced difference of the holding current *Δ*^*S*^*I*_hc_ was (-) 0.17 nA ((-) 0.19 ± 0.01 nA, n = 14) at − 45 mV. The minus sign (-) emphasizes that cell stretch leads to more negative values of the initial net holding current at the level of *V*_hp_ = − 45 mV. The pulse to 0 mV induced the *L-type Ca*^*2+*^ current (*I*_Ca,L_) that activated and inactivated over time. The stretch attenuated the *L-type Ca*^*2+*^ current (Fig. 3A.1), which was estimated as the difference between the negative peak current *I*_max_ and the current at the end of the pulse in control (*I*_L_) (from |1.55| nA to |1.11| nA during the stretch). The module for *I*_Ca,L_ was used because the current value was calculated as a distance (Fig. 3A.1, B.2, C.2) from the starting point (the value of *I*_Ca,L_ on the background of nifedipine = *I*_L_, in the positive region) to the point *I*_max_, in the negative region. The time course of the stretch - induced difference of the *L-type Ca*^*2+*^ current in the control and during the stretch (*Δ*^*S*^*I*^*tc*^_Ca,L_) was (+) 0.44 nA ((+) 0,44 ± 0.07 nA, n = 7). The plus sign indicates that cell stretching at the *V*_hp_ = − 45 mV led to a shift of negative *I*_Ca,L_ to more positive values compared to the initial values, resulting in a decrease in the negative area.

The *I/V* curves in Fig. 3A.2 show the voltage dependence of *I*_L_ and *I*_Ca,L_, and their modulation by a 6 μm stretch. Before stretching, the *I*_L_ - *I/V* curve was *N*-shaped and crossed the voltage axis (zero current potential *V*_0_) at − 78 mV (− 75 ± 3 mV, n = 7; equivalent to the resting potential of the non-clamped cell). The modest stretch shifted the net currents *I*_L_ to more negative values: *Δ*^*S*^*I*_L(− 45)_ is (-) 0.15 nA ((-) 0.16 ± 0.02 nA, n = 7) at − 45 mV and *Δ*^*S*^*I*_L(−80)_ is (-) 0.24 nA ((-) 0.26 ± 0.03 nA, n = 7) at − 80 mV. The *V*_0_ also shifted to -70 mV (− 66 ± 3 mV, n = 7) with the stretch.

During cell stretching, although the changes in the ^*S*^*I*_L(− 45)_ current occur in the positive range, their values are lower than the control values of *I*_L(−45)_. The minus (-) sign of *Δ*^*S*^*I*_L(−45)_ emphasizes that cell stretch leads to a change in the curve toward more negative values. Similarly, for *I*_L(−80)_, which is usually in the negative range, cell stretch further increases this negative current ^*S*^*I*_L(−80)_, leading to a negative differential current *Δ*^*S*^*I*_L(−80)_ that only shows the direction of its change. The *I/V* curves recorded before and during the stretch crossed each other close to 0 mV, and at positive potentials, the late current increased with the stretch. The *I/V* curve of *I*_Ca,L_ decreased during the 6 μm stretch by *Δ*^*S*^*I*_Ca,L_ = (+) 0.38 nA ((+) 0.32 ± 0.04 nA, n = 7) compared to the control values.

Upon stretching to 8 μm, the holding current ^*S*^*I*_hc_ decreased to values close to 0 (label S, Fig. 3B.1), compared to the control holding current (label C, the beginning of the blue tracesin this figure). In this case, the stretch-induced difference in the holding current *Δ*^*S*^*I*_hc_ was (-) 0.41 nA ((-) 0.42 ± 0.02 nA, n = 7) at *V*_hp_ = − 45 mV. The pulse at 0 mV induced *I*_Ca,L_, and the stretch attenuated *I*_Ca,L_ (Fig. 3B.1, negative current wave from blue in control to red during the stretch) from |1.55| nA in control to |0.94| nA. During time course registration, the stretch-induced difference of *L-type Ca*^*2+*^ current in control and during the stretch, *Δ*^*S*^*I*^*tc*^_Ca,L_ equals (+) 0.61 nA ((+) 0,60 ± 0.04 nA, n = 7).

Stretching by 8 μm shifted the *I/V* relation to more negative currents than a 6 μm stretch (Fig. 3B.2): At − 45 mV, the stretch-induced difference in current was *Δ*^*S*^*I*_L(−45)_ = (-) 0.29 nA ((-) 0.37 ± 0.06 nA, n = 7), while at − 80 mV, it was *Δ*^*S*^*I*_L(−80)_ = (-) 0.57 nA ((-) 0.60 ± 0.09 nA, n = 7), and *V*_0_ was depolarized to -56 mV (− 54 ± 3 mV, n = 7). The *I/V* curve of *I*_Ca,L_ during the stretch showed a further decrease in ^*S*^*I*_Ca,L_, resulting in an increase in the differential current *Δ*^*S*^*I*_Ca,L_ to (+) 0.52 nA ((+) 0.52 ± 0.02 nA, n = 7).

The maximum stretch of 10 μm caused a shift in the holding current ^*S*^*I*_hc_ to the negative region with values equal to − 0.16 nA (label S, the beginning of the red traces in Fig. 3C.1, compared to label C, the beginning of the blue traces). The stretch-induced difference in the holding current *Δ*^*S*^*I*_hc_ was (-) 0.61 nA ((-) 0.74 ± 0.10 nA, n = 7) at − 45 mV. This stretch resulted in a further reduction of *I*_Ca,L_ (Fig. 3C.1: negative current wave from blue in control to red during the stretch) from |1.55| nA in control to |0.77| nA. When registering the time course, *Δ*^*S*^*I*^*tc*^_Ca,L_ was equal to (+) 0.78 nA ((+) 0.77 ± 0.04 nA, n = 6).

Stretching the cell by 10 μm caused a larger shift in the *I/V* relationship to more negative currents compared to an 8 μm stretch (Fig. 3C.2): *Δ*^*S*^*I*_L(−45)_ was (-) 0.57 nA ((-) 0.71 ± 0.09 nA, n = 6) at − 45 mV, while *Δ*^*S*^*I*_L(−80)_ was (-)1.33 nA ((-)1.41 ± 0.15 nA, n = 6) at − 80 mV, and depolarized *V*_0_ to − 35 mV (− 39 ± 3 mV, n = 6). During this stretch, the *I/V* curve of *I*_Ca,L_ showed a larger decrease in ^*S*^*I*_Ca,L_, resulting in an increased differential current *Δ*^*S*^*I*_Ca,L_ of (+) 0.66 nA ((+) 0.65 ± 0.05 nA, n = 6).

In K^+^ _in_/K^+^ _out_ solutions, Gd^3+^ eliminates stretch-induced reduction in *I*_Ca,L_ at all levels of cell stretch, as previously demonstrated [5, 6].

**Fig. 4:**
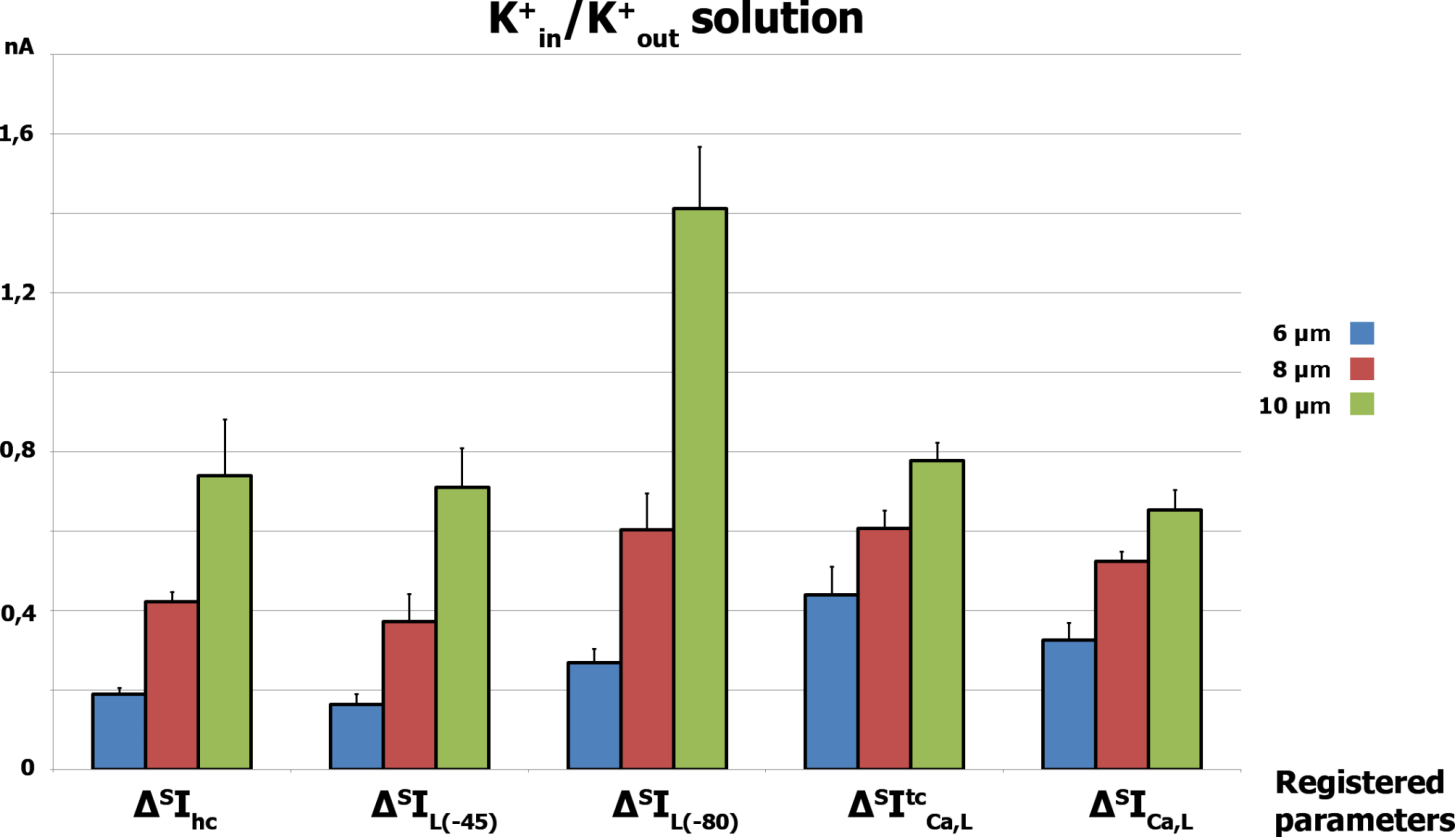
Comparison of the mean values of the differential currents *Δ*^*S*^*I*_hc_, *Δ*^*S*^*I*_L(−45)_, *Δ*^*S*^*I*_L(−80)_, *Δ*^*S*^*I*^*tc*^_Ca,L_ and *Δ*^*S*^*I*_Ca,L_ during cell stretching at 6, 8, and 10 μm in K^+^ _in_/K^+^ _out_ solutions. Error bars represent ± SD for n experiments (n = 7)

The comparison of the mean values of differential currents *Δ*^*S*^*I*_hc_, *Δ*^*S*^*I*_L(−45)_, *Δ*^*S*^*I*_L(−80)_, *Δ*^*S*^*I*^*tc*^_Ca,L_ and *Δ*^*S*^*I*_Ca,L_ during cell stretch at 6, 8, and 10 μm is presented in Fig. 4. It should be noted that *Δ*^*S*^*I*_hc_, *Δ*^*S*^*I*_L(−45)_, and *Δ*^*S*^*I*_L(−80)_ are mechanosensitive and are based on the activation of stretch-activated channels (SAC).

### **Local stretch increased current through nonselective cation channels*I***_**ns**_**and, at the same time, reduced*I***_**Ca,L**_**in Cs**^**+**^_**in**_**/Cs**^**+**^_**out**_**solutions (time course and voltage dependence).**

**Fig. 5:**
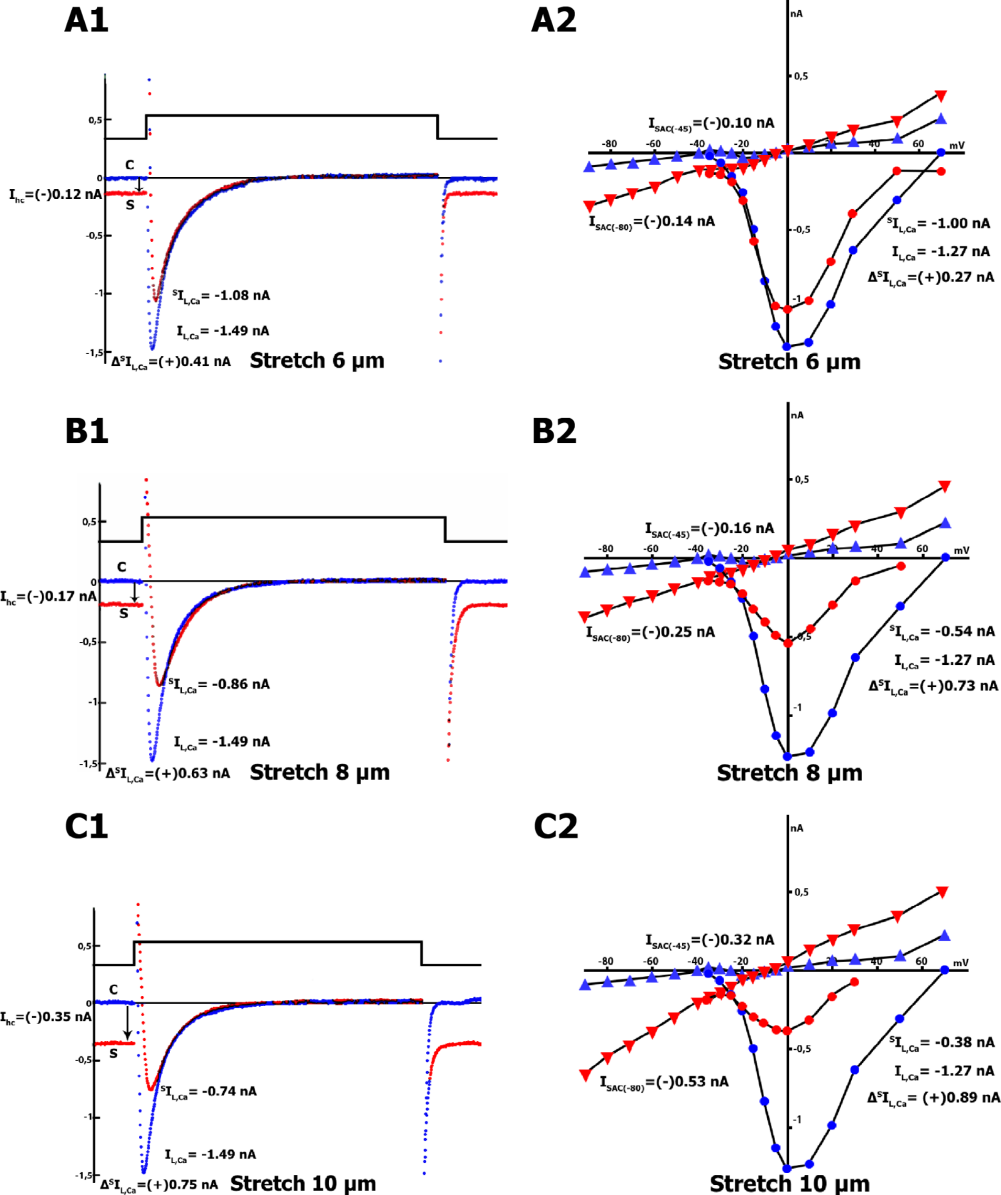
Reduction of *I*_Ca,L_ in Cs^+^ _in_/Cs^+^ _out_ solutions with *K*^*+*^ currents suppressed during local stretching of cardiomyocytes by 6, 8, and 10 μm. *V*_hp_ = − 45 mV. **A**: (6 μm stretch). **A.1** – The time course of the membrane current. The holding current at *V*_hp_ in control (beginning of the blue traces - label C) and during stretching (beginning of the red traces – label S). A pulse from − 45 to 0 mV induces *I*_Ca,L_, which decreases during stretching (indicated by a negative blue current wave compared to a negative red wave). **A.2** – *I/V* curve of *I*_ns_ before (blue triangles) and during (red triangles) stretching, as well as *I*_Ca,L_ before (blue circles) and during (red circles) stretching. **B**: (8 μm stretch). **B.1** - The time course of the membrane current before and during stretching, which results in a greater reduction of *I*_Ca,L_ compared to **A.1**. Notations as in A.1. **B.2** – *I/V* curves of *I*_ns_ and *I*_Ca,L_ before and during stretching. Notations as in A.2. **C**: (10 μm stretch). **C.1** – The time course of the membrane current in control and during stretching. *I*_Ca,L_ decreases with increasing stretching. Notations as in A.1. **C.2** – *I/V* curves of *I*_ns_ and *I*_Ca,L_ before and during stretching. Notations as in A.2

In Fig. 5A.1, the effect of 6 μm stretch on time-dependent membrane currents in Cs^+^ _in_/Cs^+^ _out_ solutions is presented. At the control stage, the holding current was 0 nA (labeled as C, Fig. 5A.1), while stretch resulted in negative holding current values (labeled as S, Fig. 5A.1). The difference in the holding current induced by stretch was (-) 0.12 nA (0.09 ± 0.01 nA, n = 7) at − 45 mV. The pulse to 0 mV resulted in an *L-type Ca*^*2+*^ current of − 1.49 nA (− 1.62 ± 0.13 nA, n = 18), or − 7.3 pA/pF (− 7.1 ± 0.2 pA/pF, n = 18). The stretch of 6 μm reduced the *L-type Ca*^*2+*^ current, ^S^*I*_Ca,L_ (as shown by the negative current wave in Fig. 5A.1, from blue to red). During the recording of the time course, the stretch-induced difference of *L-type Ca*^*2+*^ current in control and during the stretch (*Δ*^*S*^*I*^*tc*^_Ca,L_) was (+) 0.41 nA ((+) 0.35 ± 0.05 nA, n = 7).

Figure 5 A.2 displays the voltage dependence of *L*_ns_, *I*_Ca,L_ and its modulation by a 6 μm stretch, as shown in the *I/V* curves. Before stretching, the *I/V* curve of *I*_ns_ was linear and intersected the voltage axis (zero current potential *V*_0_) at − 40 mV (− 38 ± 3 mV, n = 7). After a modest 6-µm stretch, *I*_ns_ shifted to more negative values, and the resulting *I*_SAC_ = ^*S*^*I*_ns_ - ^*C*^*I*_ns_ (in Cs^+^ _in_/Cs^+^ _out_ solutions) was (-) 0.10 nA ((-) 0.09 ± 0.02 nA, n = 7) at − 45 mV and (-) 0.14 nA ((-) 0.12 ± 0.01 nA, n = 7) at -80 mV, while *V*_*0*_ changed to − 5 mV (− 6 ± 3 mV, n = 7). At potentials close to 0 mV, the *I/V* curves recorded before and during the stretch intersected, while at positive potentials, *I*_ns_ exhibited an increase due to the stretch. Furthermore, the *I/V* curve of ^*S*^*I*_Ca,L_ during the stretch decreased from − 1.27 nA in the control to − 1.00 nA, resulting in a *Δ*^*S*^*I*_Ca,L_ of (+) 0.27 nA ((+) 0.34 ± 0.06 nA, n = 7) when compared to the control values.

When subjected to an 8 μm stretch, the difference in the holding current at − 45 mV changed to values close to (-) 0.17 nA ((-) 0.16 ± 0.301 nA, n = 7; labeled S, Fig. 5B.1, compared to label Cin the figure). The pulse from − 45 to 0 mV induced *I*_Ca,L_, but the 8 μm stretch attenuated ^*S*^*I*_Ca,L_ (as seen in Fig. 5B.1, where the negative current wave shifts from blue to red during the stretch), from − 1.49 nA in the control to − 0.86 nA. When the time course was registered, *Δ*^*S*^*I*^*tc*^_Ca,L_ was calculated to be (+) 0.63 nA ((+) 0.64 ± 0.06 nA, n = 7).

The 8 μm stretch caused a shift in the *I/V* relation to more negative currents compared to the 6 μm stretch (as seen in Fig. 5B.2): *I*_SAC(−45)_ was (-) 0.16 nA ((-) 0.16 ± 0.02 nA, n = 7) at − 45 mV, and *I*_SAC(−80)_ was (-) 0.25 nA ((-) 0.18 ± 0.04 nA, n = 7) at − 80 mV. During this stretch, the *I/V* curve of *I*_Ca,L_ further decreased ^*S*^*I*_Ca,L_ from − 1.27 to − 0.54 nA, resulting in an increase in differential current *Δ*^*S*^*I*_Ca,L_ to (+) 0.73 nA ((+) 0.62 ± 0.09 nA, n = 7) compared to control values.

The longest 10 μm stretch caused a shift of the holding current to the negative region (label S, Fig. 5C.1, compared to label C). The stretch-induced difference in the holding current was (-) 0.35 nA ((-) 0.34 ± 0.02 nA, n = 7) at − 45 mV. Furthermore, this stretch reduced ^*S*^*I*_Ca,L_ even more (Fig. 5C.1), from − 1.49 nA in the control to − 0.74 nA. In the case of time course registration, *Δ*^*S*^*I*^tc^_Ca,L_ equals (+) 0.75 nA ((+) 0.75 ± 0.05 nA, n = 7).

Stretching by 10 μm shifted the *I/V* relation to more negative currents than an 8 μm stretch (Fig. 5C.2). Specifically, *I*_SAC(−45)_ was (-) 0.32 nA ((-) 0.34 ± 0.05 nA, n = 7) and *I*_SAC(−80)_ was (-) 0.53 nA ((-) 0.48 ± 0.10 nA, n = 7). The 10-µm stretch further reduced ^*S*^*I*_Ca,L_ to − 0.38 nA (Fig. 5C.2, red circles), resulting in an increase in differential current *Δ*^*S*^*I*_Ca,L_ to (+) 0.89 nA ((+) 0.79 ± 0.09 nA, n = 7).

In Cs^+^ _in_/Cs^+^ _out_ solutions, Gd^3+^ eliminates the reduction of stretch-induced *I*_Ca,L_ as well as *Δ*^*S*^*I*_L(−45)_, *Δ*^*S*^*I*_L(−80)_, as previously demonstrated in our studies [5, 6] (not shown in Fig. 5).

**Fig. 6:**
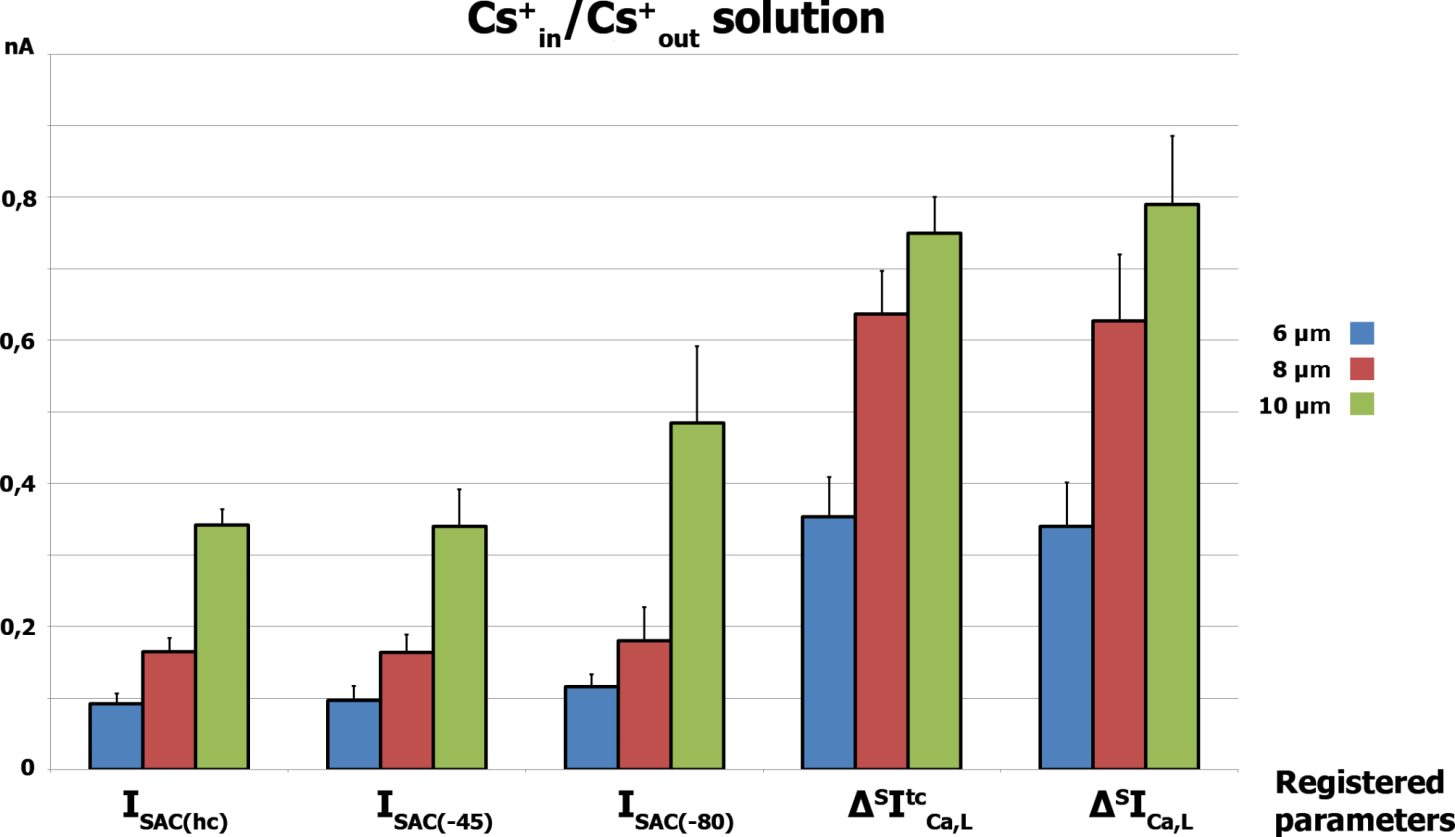
Comparison of the mean values of the differential currents *I*_SAC(hc)_, *I*_SAC(−45)_, *I*_SAC(−80)_, *Δ*^*S*^*I*^*tc*^_Ca,L_ and *Δ*^*S*^*I*_Ca,L_ during cell stretching at 6, 8, and 10 μm in Cs^+^ _in_/Cs^+^ _out_ solutions. The ± SD is shown for *n* experiments (n = 7)

Figure 6 compares the mean values of *I*_SAC(hc)_, *I*_SAC(−45)_, *I*_SAC(−80)_, ^*S*^*I*_Ca,L_, and *Δ*^*S*^*I*_Ca,L_ during cell stretching at 6, 8, and 10 μm in Cs^+^ _in_/Cs^+^ _out_ solutions. *I*_SAC(hc)_, *I*_SAC(−45)_, and *I*_SAC(−80)_ are also mechanosensitive currents based on the work of SAC. Additionally, *I*_Ca,L_ is likely to be conducted through Ca_V_1.2 channels and is also mechanosensitive in ventricular rat cardiomyocytes.

### **Gd**^**3+**^**eliminates the*L-type Ca***^***2+***^**current via Ca**_**V**_**1.2 channels in K**^**+**^_**in**_**/K**^**+**^_**out**_, **Cs**^**+**^_**in**_**/K**^**+**^_**out**_, **K**^**+**^_**in**_**/Cs**^**+**^_**out**_**and Cs**^**+**^_**in**_**/Cs**^**+**^_**out**_**solutions**

**Fig. 7:**
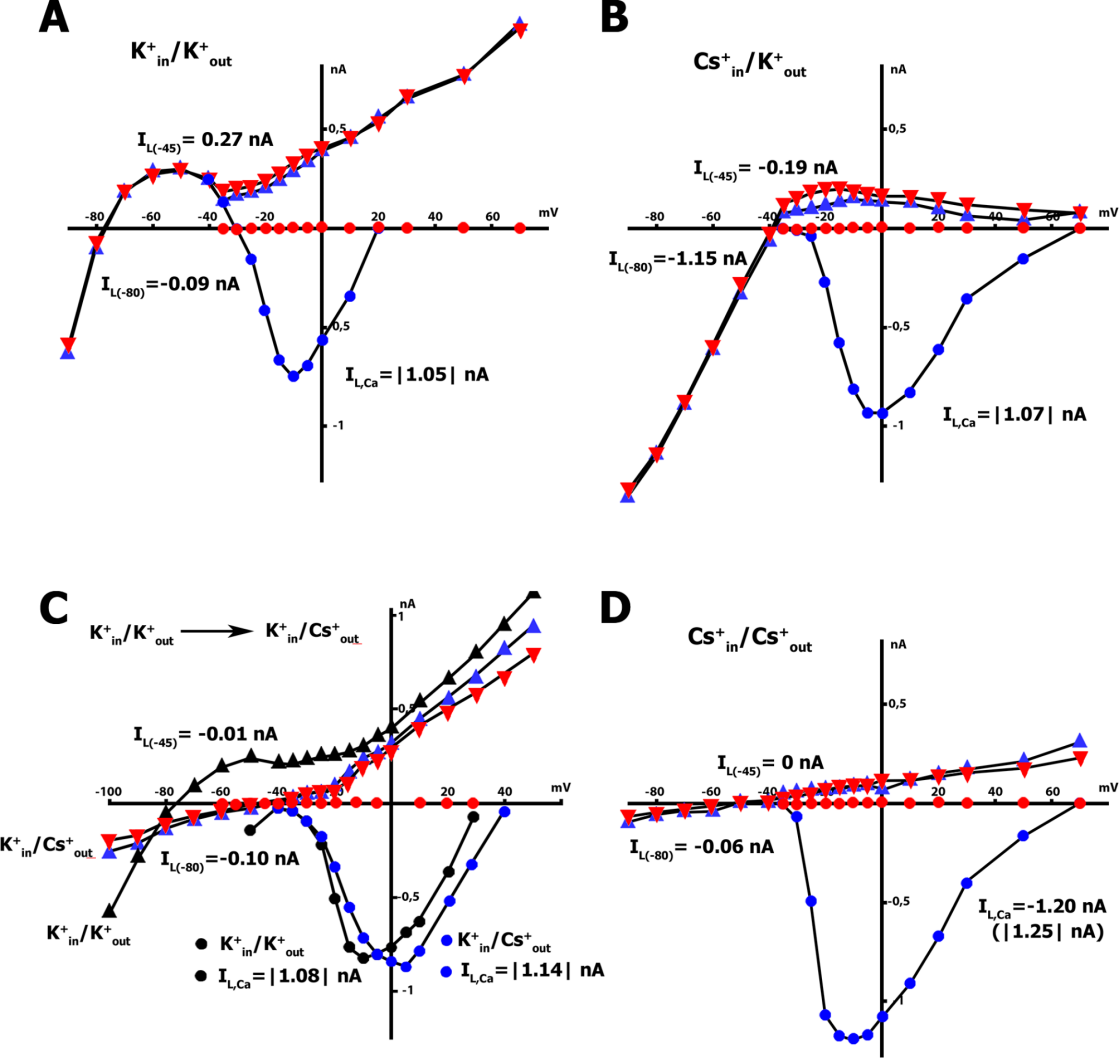
Effect of 5 µM Gd^3+^ on *I*_L_ and *I*_Ca,L_ in K^+^ _in_/K^+^ _out_ (**A**), Cs^+^ _in_/K^+^ _out_ (**B**), K^+^ _in_/Cs^+^ _out_ (**C**), and Cs^+^ _in_/Cs^+^ _out_ (**D**) solutions. Control curves in blue: *I*_L_ – triangles and *I*_Ca,L_ – circles. Curves in the presence of Gd^3+^ in red: *I*_L_ – triangles and *I*_Ca,L_ – circles. Note: C: Switching cell perfusion from K^+^ _in_/K^+^ _out_ solution (*I*_L_ – black triangles and *I*_Ca,L_ – black circles) to K^+^ _in_/Cs^+^ _out_ solution (*I*_L_ – blue triangles and *I*_Ca,L_ – blue circles), followed by the application of Gd^3+^ (*I*_L_ – red triangles and *I*_Ca,L_ – red circles). While Gd^3+^ exerts only a minor effect on *I*_L_, it eliminates *I*_Ca,L_ in all solutions tested

Figure 7. A, B, C, and D illustrate that *I*_Ca,L_ values remained constant (1.48 ± 0.06 nA, n = 24) under all experimental conditions tested, including K^+^ _in_/K^+^ _out_, Cs^+^ _in_/K^+^ _out_, K^+^ _in_/Cs^+^ _out,_ and Cs^+^ _in_/Cs^+^ _out_ solutions. Furthermore, during the transition of cell perfusion from K^+^ _in_/K^+^ _out_ to K^+^ _in_/Cs^+^ _out_ solutions (Fig. 7C), *I*_Ca,L_ values did not change (n = 6). The *I*_Ca,L_ values were determined by calculating the maximum calcium peak current at a given potential at the point on the late current curve that corresponds to that potential. For Cs^+^ _in_/Cs^+^ _out_ solutions, two variants of *I*_Ca,L_ values were presented (Fig. 7B): (1) up to zero and (2) up to a characteristic point on the late current curve. Typically, only the value (1) is used for calculations in the literature.

It has been shown that, on the background of cell stretch, Gd^3+^ at a concentration of 5 µM blocks *I*_SAC_ [6]. The addition of Gd^3+^ abolished the dependence of *I*_SAC_ on all local stretch values. Our experiments revealed that Gd^3+^ also had a minor inhibitory effect on the *I*_L_ current at − 45 and − 80 mV, suggesting that *I*_SAC_ may have contributed to the net currents of the non-stretched cells. This is depicted in Fig. 7, where the blue triangles indicate the control and the red triangles indicate the application of the blocker (n = 6). Additionally, Gd^3+^ did not affect the background current, *I*_*K1*_ (Fig. 7A).

To investigate potential changes in *L-type Ca*^*2+*^ current through Ca_V_1.2 channels, we applied Gd^3+^, which is known to be a nonspecific blocker of the mechanically gated channel’s current *I*_SAC_. Our results indicated that the addition of 5 µM of Gd^3+^ eliminated *L-type Ca*^*2+*^ current in K^+^ _in_/K^+^ _out_ (n = 6), Cs^+^ _in_/K^+^ _out_ (n = 6), K^+^ _in_/Cs^+^ _out_ (n = 6), and Cs^+^ _in_/Cs^+^ _out_ (n = 6) solutions (blue circles in the control compared to red circles after application of the blocker).

## Discussion

### Channel transcripts and their mechanosensitivity

In ventricular cardiomyocytes, mechanosensitive channels are believed to play a role in the regulation of the contractile properties of the heart [5]. In our experiments, we found significant amounts of transcripts for the mechanosensitive channels TRPM7, TRPC1, and TRPM4 (Table 1).

TRPM7 is a nonselective cation channel that is permeable to both Ca^2+^ and Mg^2+^ [102]. It has been shown to contribute to the stretch-induced current in ventricular cardiomyocytes by mediating a Ca^2+^ influx in response to mechanical stretch [103, 104]. This influx of Ca^2+^ is believed to activate downstream signaling pathways that regulate contractility [103]. TRPC1 was shown to contribute to the stretch-induced current in ventricular cardiomyocytes by mediating a non-selective cation influx in response to mechanical stretch, which influx is believed to depolarize the cell membrane and contribute to the regulation of contractility [104]. TRPM4 is a Ca^2+^-activated non-selective cation channel that has been shown to contribute to the stretch-induced current in ventricular cardiomyocytes [45]. It is believed to be activated by Ca^2+^ influx through other channels such as TRPC1 and TRPM7 [37, 42, 105]. TRPM4 activation is believed to contribute to contractility regulation by modulating the duration of AP [106].

Studies on mechanosensitivity in murine ventricular myocytes have shown a link between stretch-activated TRPC6 channels as well as stretch-deactivated GK1 and especially Kir2.3 channels [22]. GK1 channels are formed by Kir2.1, Kir2.2, and Kir2.3 proteins [107, 108]. In our experiments, we found multiple transcripts of Kir2.1 and Kir2.2 in isolated ventricular myocytes from rats, while Kir2.3 was absent. Therefore, in K^+^ _in_/K^+^ _out_ solutions, the mechanosensitivity of ventricular myocytes is probably determined by the stretch-activated TRPM7, TRPC1, and TRPM4 channels and the stretch-deactivated GK1 (Kir2.1 and Kir2.2) channels. Our findings are consistent with other studies [109], which have shown that ventricular *I*_K1_ is based primarily on the heteromeric assembly of the Kir2.1 and Kir2.2 channels, while the Kir2.3 channels are more relevant in the atrium.

### **The local stretch of cells modulates*I***_**Ca,L**_, **probably through Ca**_**V**_**1.2 channels**

One of the main questions of the study was to determine the cause of changes in *I*_Ca,L_. Our observations showed a decrease in ^*S*^*I*_Ca,L_ with discrete cell stretching, while there was a corresponding increase in *Δ*^*S*^*I*^*tc*^_Ca,L_ and *Δ*^*S*^*I*_Ca,L_ during cell stretching at 6, 8, and 10 μm.

In general, membrane channels (including voltage-gated) are expected to respond to mechanical stimuli because mechanical energy can affect the energy barriers between different conformational states of a channel protein [110]. For example, longitudinal stretching has been shown to modify the gating mechanism of; Na_V_ channels [111], such as Na_V_1.5 [81, 82] and Na_V_1.6 [83], Ca_V_ channels such as Ca_V_1.2 [84], Ca_V_1.3 [85], and Ca_V_2.2 channels [86], as well as K_V_ channels such as K_V_1.2 [67], K_V_1.5, and K_V_3.2 [87, 112]. In addition, mechanical energy can affect the conformation of proteins associated with cytoskeletal elements [113, 114], such as F-actin [114] and integrins [115]. This can also activate stretch-sensitive kinases (Src, MAP) or phosphatases, which can, in turn, modulate the channel protein by phosphorylation and dephosphorylation [109]. Finally, mechanical stretch can also impact the production rate of reactive oxygen species, or NO, which can modify channel gating through oxidative or nitrosative mechanisms [116], which in turn can play a role in stretch-induced effects on Ca^2+^ release from the sarcoplasmic reticulum (SR) [116, 117].

The *L-type Ca*^*2+*^ current is responsible for generating the negative current wave observed at the start of the depolarizing clamp step (Fig. 3A.1, B.1, and C.1), as well as the long-lasting plateau of the AP. Interestingly, the local mechanical stretch has been shown to decrease *I*_Ca,L_, with the reduction being observed across the entire range of clamp potentials. In particular, the voltage dependence of *I*_Ca,L_ appears to remain unchanged under these conditions (see Fig. 3A.2, B.3, and C.4).

In ventricular myocytes, some nonselective channels are known to be activated by an increase in cytosolic calcium concentration ([Ca^2+^]_C_) [118]. To investigate whether stretch-induced changes in [Ca^2+^]_C_ might play a role in these effects, Gannier et al. (1996) conducted experiments in which they prevented possible stretch-induced increments in [Ca^2+^]_C_ [119]. Interestingly, they have found that chelation of the [Ca^2+^]_C_ does not have a significant effect on *I*_SAC_. However, we observed significant effects of BAPTA on the stretching effects on *I*_Ca,L_. Specifically, while *I*_SAC_ in Cs^+^ _in_/Cs^+^ _out_ solutions was found to be insensitive to chelation of [Ca^2+^]_C_, the stretch-induced reduction of *I*_Ca,L_ disappeared after dialyzing the cell with 5 mM BAPTA in the patch pipette [6].

Since stretch has been shown to increase [Ca^2+^]_C_ and BAPTA can chelate cytosolic Ca^2+^, it is likely that the stretch-induced reduction of ^*S*^*I*_*Ca,L*_ results from the stretch-induced increase in [Ca^2+^]_C_ followed by Ca^2+^-mediated inactivation of the Ca^2+^ channel (Ca^2+^-calmodulin interaction with the Ca^2+^ channel α subunit) [12]. We propose that the primary effect of stretch is to increase [Ca^2+^]_C_ via an increase in Ca^2+^ influx through SACs or via Ca^2+^ release from the SR in the vicinity of the L-type Ca^2+^ channel. Previous research has suggested that *I*_Ca,L_ can be reduced by an increase in [Ca^2+^]_C_ and by stimulation of *I*_P_ (current due to electrogenic sodium pumping) by an increase in [Na^+^]_C_. The concept of indirect activation by stretch should also be applied to the current generated by Na^+^/Ca^2+^-exchange if [Na^+^] is elevated by Na^+^ influx due to *I*_SAC_ [120] and to K^+^ channels activated by Ca^2+^ or Na^+^ ions [121]. Alternatively, the stretch may increase [Ca^2+^]_C_ by Ca^2+^ release from the SR [117].

Cytoskeletal F-actin fibers were found to be involved specifically in *I*_SAC_ activation and regulation of the Ca_V_1.2 channel gating mechanism. In the context of cell stretch, which has been shown to increase *I*_SAC_ and decrease *I*_Ca,L_, treatment with cytochalasin D, a toxin known to depolymerize F-actin, blocks the effects of stretch on late currents and leads to further reduction of *I*_Ca,L_ [8], which also corroborates the effect of cell stretch, probably on the Ca_V_1.2 channel.

We make such an assumption based on the analysis of the transcript quantities for Ca^2+^ ion channel genes. As we have demonstrated, the transcript counts for the Ca_V_1.2 channel gene are exceptionally high, measuring 1336.66 ± 71.8. In contrary, for Ca_V_1.3, this count is quite low at 17.33 ± 2.03, and for Ca_V_1.1, it is minimal at 0.33 ± 0.33. We did not detect other channels involved in the whole *L-type Ca*^*2+*^ current. Based on these numerical values, we can infer the primary role of the Ca_V_1.2 channel in the formation of the *L-type Ca*^*2+*^ current that we observed.

### Voltage-gated current modulation or mechanosensitivity of the (Ca_V_1.2) channel

It is now well established that Gd^3+^ blocks a range of ion channels, including SACs (mechanically gated non-selective cation channels, MGC_ns_, and mechanically gated potassium channels, MGC_K_), which was previously assumed in the study by Yang et al. (1989) [122]. In addition, Gd^3+^ has been found to block several other ion channels, such as BK_Ca_ channels cloned from chick ventricular myocytes [123] and *delayed rectifier current* (*I*_K_) in guinea pig single ventricular myocytes, including both *I*_KR_ (rapidly activated) and *I*_KS_ (slowly activated), while the background current *I*_K1_, was not affected by Gd^3+^ [124]. Moreover, Gd^3+^ was a potent *I*_Na_ blocker near the threshold potential for Na^+^ channels in rabbit ventricular myocytes [122]. It has been demonstrated that Gd^3+^ is also a potent blocker of L-type Ca^2+^ channels in isolated guinea pig ventricular myocytes [125].

In our experiments, treatment with the nonspecific inhibitor of *I*_SAC_, Gd^3+^, on the background of cell stretching eliminated both *I*_SAC_ and *I*_Ca,L_ at all stretching magnitudes and voltage steps relative to the *V*_hp_. This response to Gd^3+^ was maintained in Cs^+^ _in_/Cs^+^ _out_ solutions, indicating that voltage-gated channels, apparently Ca_V_1.2 in adult rat ventricular myocytes, have additional mechanosensitive properties.

## Conclusion

Our analysis revealed the presence of transcripts for the TRPM7, TRPC1, and TRPM4 channels that are known to exhibit mechanosensitivity. Furthermore, we detected mechanosensitive transcripts of the Kir6.2 and Kir6.1 channels, as well as transcripts of GK1 channels formed by the Kir2.1 and Kir2.2 proteins. Although the detection of the TREK-1/K_2P_2.1 transcript was limited, indirect data from other studies supports its presence [77]. The highest number of RNA reads among all Ca_V_ channels was detected for Ca_V_1.2 channels, which themselves exhibit mechanosensitivity.

Cell stretching at various magnitudes discretely increased *I*_SAC_ and decreased *I*_Ca,L_ probably through Ca_V_1.2 channels in K^+^ _in_/K^+^ _out_, Cs^+^ _in_/K^+^ _out_, K^+^ _in_/Cs^+^ _out,_ and Cs^+^ _in_/Cs^+^ _out_ solutions. However, treatment with the nonspecific blocker of *I*_SAC_, Gd^3+^, on the background of cell stretching eliminated both *I*_SAC_ and *I*_Ca,L_ at all stretching magnitudes and voltage steps relative to *V*_hp_. The study suggests that voltage-gated Ca_V_1.2 channels in adult rat ventricular myocytes have additional mechanosensitive properties, as evidenced by the maintained response to Gd^3+^ in the Cs^+^ _in_/Cs^+^ _out_ solution.

### Electronic supplementary material

Below is the link to the electronic supplementary material.

Supplementary Material 1

## Data Availability

The datasets generated during and/or analyzed during the current study are available in the following repository [https://disk.yandex.ru/d/40Xg_q8soUg7AA].
